# Perspective-taking is associated with increased discriminability of affective states in the ventromedial prefrontal cortex

**DOI:** 10.1093/scan/nsac035

**Published:** 2022-05-17

**Authors:** Anthony G Vaccaro, Panthea Heydari, Leonardo Christov-Moore, Antonio Damasio, Jonas T Kaplan

**Affiliations:** Jon Brain and Creativity Institute, Department of Psychology, University of Southern California, Los Angeles, CA 90089-0001, USA; Jon Brain and Creativity Institute, Department of Psychology, University of Southern California, Los Angeles, CA 90089-0001, USA; Jon Brain and Creativity Institute, Department of Psychology, University of Southern California, Los Angeles, CA 90089-0001, USA; Jon Brain and Creativity Institute, Department of Psychology, University of Southern California, Los Angeles, CA 90089-0001, USA; Jon Brain and Creativity Institute, Department of Psychology, University of Southern California, Los Angeles, CA 90089-0001, USA

**Keywords:** emotion, perspective-taking, multivariate-pattern analysis, ventromedial prefrontal cortex

## Abstract

Recent work using multivariate-pattern analysis (MVPA) on functional magnetic resonance imaging (fMRI) data has found that distinct affective states produce correspondingly distinct patterns of neural activity in the cerebral cortex. However, it is unclear whether individual differences in the distinctiveness of neural patterns evoked by affective stimuli underlie empathic abilities such as perspective-taking (PT). Accordingly, we examined whether we could predict PT tendency from the classification of blood-oxygen-level-dependent (BOLD) fMRI activation patterns while participants (*n* = 57) imagined themselves in affectively charged scenarios. We used an MVPA searchlight analysis to map where in the brain activity patterns permitted the classification of four affective states: happiness, sadness, fear and disgust. Classification accuracy was significantly above chance levels in most of the prefrontal cortex and in the posterior medial cortices. Furthermore, participants’ self-reported PT was positively associated with classification accuracy in the ventromedial prefrontal cortex and insula. This finding has implications for understanding affective processing in the prefrontal cortex and for interpreting the cognitive significance of classifiable affective brain states. Our multivariate approach suggests that PT ability may rely on the grain of internally simulated affective representations rather than simply the global strength.

## Introduction

Contemporary neuroscience research has highlighted the complex relationship between neural activity and affective states. We define ‘affective states’ to include both ‘emotions’, which in our view comprise physiological and motoric responses to stimuli in the environment that are relevant to the homeostatic welfare of the organism, and ‘feelings’, the conscious perceptions of emotion-related changes in the body. Affective states appear to involve interactions between cortical and sub-cortical regions, as well with the viscera (for review: Kober *et al.*, 2008; Critchley, 2009; Tettamanti *et al.*, 2012; Damasio and Carvalho, 2013; Smith and Lane, 2015; Vaccaro *et al.*, 2020).

Recently, our understanding of emotion has progressed from considering solely the experience and mechanisms of individual experience, understanding that the consideration of other’s minds plays a large role in one’s own affective experience (for review: Decety and Meltzoff, 2011; Christov-Moore and Iacoboni, 2016; Lamm *et al.*, 2016). While the core mechanisms of affect were once viewed and researched as a primarily private, intraindividual process, there is a growing consensus that the development of affect is inescapably linked to sociality: this places empathetic processes as more core to individual affect than they were originally considered (for review: Parkinson and Manstead, 2015; Fotopoulou and Tsakiris, 2017; Dukes *et al.*, 2021). Empathy is a multifaceted construct combining cognitive processes that allow us to understand the internal states of others and affective processes that allow us to share in the internal states of others. These include aversive reactions to others’ distress [personal distress (PD)], concern for others’ welfare [empathic concern (EC)], feeling and understanding the experiences of hypothetical or absent others (fantasizing) and taking others’ perspectives [perspective-taking (PT)] (Davis, 1983). Research has found that systems involved in understanding one’s own emotions are also involved in understanding the affective states of others (Ochsner *et al.*, 2004). Empathizing with another’s feelings recruits affective brain regions involved in representing one’s own affective state (Singer *et al.*, 2004; Lamm *et al.*, 2016) and there is evidence that impaired affective experience (as in psychopathy) may limit empathic abilities (Blair *et al.*, 2002). For example, participants administered an analgesic were impaired in their ability to recognize and respond to others’ pain (Mischkowski *et al.*, 2019). Furthermore, it has been found that placebo analgesia reduces both pain and empathy for pain (Rütgen *et al.*, 2015), as well as both unpleasant touch and empathy for it through modulation of the insular cortex (Rütgen *et al.*, 2021).

One feature of affective experience that is relevant to both empathizing with others and representing one’s own state is the extent of differentiation among affective states. Previous studies have shown that individuals who are more successful at judging the affective states of others experience more differentiated categories of affect (Erbas *et al.*, 2016; Israelashvili *et al.*, 2019). Having, and being able to simulate, affective states that are categorically discernable may facilitate skills such as mentalizing, empathy and PT due to the perceived increase in clarity of what one is feeling and therefore what that feeling means functionally (Hill and Updegraff, 2012; Eckland *et al.*, 2018; Thompson and Boden, 2019). Therefore, we hypothesize that increased neural differentiation of affective states may be associated with greater empathy.

Recent studies using the multivariate-pattern analysis (MVPA) find that distinct affective states may be associated with specific patterns of neural activity within a network of brain regions (Scarantino, 2012; Celeghin *et al.*, 2017; Nummenmaa and Saarimäki, 2019). MVPA studies have demonstrated that discrete, induced affective states can be accurately distinguished from each other (i.e. classified) using patterns of BOLD activation in functional magnetic resonance imaging (fMRI) data (Kassam *et al.*, 2013; Kragel and LaBar, 2015; Saarimaki *et al.*, 2016; Zhou *et al.*, 2021). The most commonly studied of these states in MVPA studies are sadness, disgust, fear, happiness and, to a lesser extent, anger, all classically considered ‘basic emotions’ (Saarimaki *et al.*, 2016; Celeghin *et al.*, 2017). However, other more subtle affective states have also been studied, such as shame, envy, contempt, pride, guilt and longing (Kassam *et al.*, 2013; Kragel and LaBar, 2015; Saarimaki *et al.*, 2018), although these may be less easily classified than their more ‘basic’ cousins (Saarimaki *et al.*, 2018).

Cortical regions found to contribute most to classification accuracy in MVPA studies tend to be consistent with those found in univariate analyses of affective processing. These regions include the medial prefrontal cortex (mPFC), inferior frontal gyrus, posterior medial cortex, insula and amygdalae (Peelen *et al.*, 2010; Kim *et al.*, 2015; Saarimaki *et al.*, 2016, 2018; Sachs *et al.*, 2018). A key region involved in both judging another’s affective state and representing one’s own affective state is the mPFC (Seitz *et al.*, 2006). Specifically, the ventral areas of mPFC have been shown to play a selective role in affective PT as compared to cognitive PT (or general theory of mind; Hynes *et al.*, 2006; Corradi-Dell’Acqua *et al.*, 2014; Healey and Grossman, 2018). Interestingly, MVPA results have further suggested that some of the mechanisms involved in representing one’s own affective state overlap with the mechanisms for empathy. The insula has been shown to have shared neural representations for pain and empathy for pain (Zhou *et al.*, 2020).

Empathy is often an implicit part of paradigms used to study emotion differentiation. In order to experimentally invoke affective states inside the fMRI scanner, it is common to present subjects with affect-provoking stimuli and also to engage subjects in voluntary mental simulation. For instance, Saarimaki *et al.* (2018) used narratives that describe the lead-up to an emotional event along with a guided imagery technique to evoke 14 different affective states. It can be difficult or impractical to design stimuli that effectively induce genuine affect. Tasks often involve explicitly asking participants to imagine themselves in emotional scenarios based on visual or audio imagery. Paradigms such as this require participants to access their concepts of emotion. Interestingly, this naturally creates individual variability where some individuals can easily generate strong feelings from retrieving emotional concepts while others cannot. This difference in the ability or motivation to deliberately simulate affective states from concepts is similar to what has been proposed in the somatic marker hypothesis for the vmPFC, generating feelings ‘as-if’ one is in a scenario (Damasio, 1996). It is possible that this individual variability presents itself in the distinctiveness of the neural states evoked in response to different cues. In line with previous work on the overlap between empathy and the representation of one’s own affective states, a trait level measure of empathy may be associated with these differences.

In our study, participants underwent an affect induction paradigm in which they viewed pictures of situations invoking fear, happiness, sadness and disgust, alongside captions describing the scenario from a first-person perspective. It is likely that this type of paradigm evokes both emotions and feelings; thus, it allows us to investigate the correlates of affective states as a whole but not to differentiate between neural patterns for emotion and for feeling. We ran two sets of MVPA analyses on the evoked neural data to investigate the classification accuracy of emotions from patterns of the fMRI activity. In the first, we examined which regions’ activation was most informative for classifying the four evoked affective states. We hypothesized that the mPFC would have the highest classification accuracy. In the second analysis, we attempted to predict individual differences in empathic ability from the classification accuracy of participants’ patterns of neural activation during emotion induction. We hypothesized that individual differences in empathic ability would be reflected in the distinctiveness of neural patterns of activation evoked by different emotions. Given the mPFC’s prominence in MVPA studies of emotions, as well as its role in affective PT, we hypothesized that classification accuracy for emotions in this region would show the highest correspondence with empathic abilities. Note that our predictions concerned empathic ability in general; since we did not hypothesize which specific components of empathy would correlate with the distinctiveness of neural patterns, our analysis was exploratory with respect to the empathy sub-scales.

## Methods

Healthy adult participants (*n* = 57) were recruited as part of two different studies, all recruited through flyers from the University of Southern California and surrounding Los Angeles area. Thirty-six participants’ data (18 female, age = 24.21 ± 8.68, range = 18–52) were collected in the first study. In a second study, 21 more participants’ data (11 female, age = 22.67 ± 6.45, range = 18–42) were collected. Since the two studies used the same experimental paradigm and stimuli, with slight differences detailed below, the data were combined to increase statistical power. All participants were right-handed, had normal or corrected-to-normal vision, no history of neurological or psychiatric conditions, All participants gave informed consent in accordance with the institutional review board approval guidelines approved by the University of Southern California. Because the second study involved a more comprehensive battery of additional behavioral measures not used in the analyses of this paper, behavioral data for those participants was collected on a different day. For this reason, 2 of the 21 participants did not provide behavioral data collected before the university shutdown due to coronavirus disease (COVID-19), leaving us with 19 participants from this second study for analyses relating to the empathy measures (9 female, age = 22.32 ± 6.14, range = 18–42): a total of 55 participants between the two studies for this second analysis.

### Interpersonal reactivity index

Behavioral measures of empathy were acquired through the Interpersonal Reactivity Index (Davis, 1983). This self-report measure consists of four seven-item sub-scales: (i) PT: the ability of the participant to take on the point of view of another individual (for example: ‘Before criticizing somebody, I try to imagine how I would feel if I were in their place’), (ii) fantasy (FS): the tendency of the participant to identify with fictitious characters (for example: ‘I really get involved with the feelings of characters in a novel’), (iii) EC: the presence of the participant’s feeling of compassion or concern for others (for example: ‘I am often quite touched by things I see happen’ and (iv) PD: the presence of the participant’s feeling of discomfort or anxiety for others (for example: ‘When I see someone who badly needs help in an emergency, I go to pieces’; Davis, 1983). For each participant, each sub-scale score was assessed separately, resulting in four distinct scores per participant.

### Stimuli

Stimuli were presented as one photo in the center of the screen with anecdotal descriptive text underneath each photo. Photos were first gathered from a subset of images in the International Affective Pictures Set (IAPS; Bradley and Lang, 2007) covering the affective categories of happiness, fear, sadness and disgust. Text sentences from the Affective Norms for English Text (ANET; Bradley and Lang, 2007) were also chosen in these four categories. Stimuli captions are written in the second person, telling the subject what they were experiencing (example: ‘As you leave the concert, a drunk vomits all over your jacket, soaking it.’). Pictures from the IAPS were then matched with a corresponding piece of text from the ANET that described a situation associated with the picture. For example, a picture of a snarling dog was combined with the caption ‘The dog strains forward, snarling and suddenly leaps out at you’ (See Supplementary Table S1 for examples).

For pictures that did not have appropriately matching text from ANET or text that did not have appropriately matching images from the IAPS, text/images were written to fit or acquired from the web. These new images were rated for valence and arousal by 51 participants in an earlier study (Supplementary Tables S2 and S3). Subjects were also asked to indicate what category of affective state each photo/text combination corresponded to. For every stimulus selected for the study, the expected category (among happy, sad, fear, disgust and neutral) was the most commonly picked category by the subjects (see Supplementary Figure S1). In addition to these emotional stimuli, non-emotional/neutral stimuli were used as a control and a fixation cross was used as a rest. Since our goal was to predict emotional states with MVPA, the analysis of the neutral images is not presented here.

## Functional neuroimaging: fMRI

### fMRI design

Stimuli were presented in an event-related design using MATLAB’s Psychtoolbox. In study 1, 60 stimuli (photo + text) were randomly presented during 4 functional runs (15 stimuli per run, 6 min per run). Each stimulus was presented for 12 s followed by a 12 s fixation cross in between each trial as a ‘rest’ period. In study 2, 45 stimuli were randomly presented during 3 functional runs. In both studies, participants were instructed to lay still, observe the displayed photograph, read the text and attempt to embody the described emotional situation as strongly as possible for each stimulus.

### fMRI data acquisition

All scanning was completed on a 3 T Siemens Prisma System Scanner at the USC Dornsife Cognitive Neuroimaging Center using a 32-channel head coil. Anatomical images were acquired with a T1-weighted magnetization-prepared rapid gradient-echo sequence (repetition time [TR]/echo time [TE] = 2300/2.26, voxel size 1-mm isotropic voxels, flip angle 9°). Functional images were acquired with a T2*-weighted gradient-echo sequence (repetition time [TR]/echo time [TE]= 2000/25 ms, 41 transverse 3-mm slices, flip angle 90°). A T2-weighted volume was acquired for blind review by an independent neuroradiologist, in compliance with the scanning center’s policy and local Institutional Review Board guidelines. T2-weighted scans were not analyzed by the researchers for any purpose in this study.

## fMRI analysis

### Preprocessing and GLM

Data were first processed using fMRI expert analysis tool, FMRIB Software Library (FSL)’s implementation of the General Linear Model (GLM; FMRIB Software Library, Smith *et al*., 2004) to generate voxel-wise *z*-statistic maps showing voxels that responded significantly to each emotion type for each participant. Those *z*-statistic maps were then used for the classification analysis. Data preprocessing was conducted in FSL (FMRIB Software Library, Smith *et al*., 2004) using brain extraction, slice-time and motion correction using motion-correction fMRIB s linear registration tool, spatial smoothing (5 mm) and high-pass temporal filtering (sigma = 50 s). The functional data were registered to each participant’s own anatomical image and the anatomical data were registered to the standard MNI Brain (Montreal Neurological Institute) using FSL’s fMRIB’s non-linear registration tool (FNIRT) tool (Jenkinson and Smith, 2001). The data were modeled with a separate regressor for each of the four emotions (happy, sad, fear and disgust), one for the neutral condition, the temporal derivatives of all task regressors and six motion parameters to account for residual motion effects. These same smoothed standard space *z*-maps were used for both the emotion discrimination analysis and for the individual subject searchlights.

## MVPA analysis

### Emotion discrimination analysis

All MPVA analyses were conducted using PyMVPA (Hanke *et al*., 2009). A whole-brain searchlight analysis was conducted to identify regions whose activation patterns allowed us to classify the four emotions across all subjects’ data. The input data to the classifier was a single 4D image file combining *z*-stat maps (normalized to MNI standard space using FNIRT) for each affective state, functional run and participant (resulting in a total of 828 concatenated images—36 participants × 4 runs × 4 emotions for study 1 combined with 21 participants × 3 runs × 4 emotions for study 2). For every voxel in the brain, a sphere centered on that voxel (radius = 5 voxels) was used to train and test a linear support vector machine (SVM) using a leave-one-out cross-validation. In other words, in each iteration, the classifier was trained on all the participants’ data except one and then tested on the remaining participant’s data leading to 55 cross-validation folds. The resulting average accuracy over all iterations, after leaving each participant out once, was mapped to the center voxel of the sphere, ultimately resulting in a cross-participant map of classification accuracies for every voxel. For the SVM regularization parameter *C*, we used the default in PyMVPA, which chooses this parameter automatically according to the norm of the data.

### Empathy correlation analysis

To correlate the scales of the Interpersonal Reactivity Index (IRI) with individual classification accuracy, we first computed whole-brain searchlights ‘within’ every individual participant’s data. We ran searchlights on each individual subject’s data in standard space. For each participant, a sphere (radius = 3 voxels) centered on every voxel in the brain was used to iteratively assess classification accuracy throughout the brain. For each sphere, the SVM classifier was trained on all but one of the emotions’ functional scanning runs and tested on the left out run. The resulting accuracy values were mapped to the center voxel of the sphere and resulting whole-brain voxel-wise accuracy maps were warped into the standard space. This created an accuracy map for each participant where a voxel’s value represented the classification accuracy of the three voxel spheres surrounding that voxel. We chose a smaller sphere size than the between-subjects analysis to increase our spatial resolution. Furthermore, these individual subject searchlights required significantly less computing power than the between-subjects analysis, so we were less restricted by computing limitations. To identify relationships between these individual participant searchlight maps and individual differences in self-reported empathy, we used FSL’s Randomise tool (with FWE correction). We created a series of regressors using participants’ demeaned scores on each of the IRI’s sub-scales (PT, EC, PD and FS). These regressors were then related to the searchlight accuracy values using Randomise’s nonparametric permutation testing approach (Winkler *et al.*, 2014). At each voxel, the accuracies across subjects are randomly associated with the regressor-values (the empathy sub-scales) and a test statistic is computed. This permutation procedure is repeated 5000 times to generate a null distribution. One of the resulting maps is then a map of 1 minus the *P*-value for the association between the regressor and the classification accuracy, determined by comparison to the null distribution. These maps were corrected for family-wise error using the FSL’s threshold-free cluster enhancement algorithm. We restricted our interpretation of the resulting maps to regions that significantly predicted emotions in the group searchlight analysis by masking these maps with the regions found to significantly distinguish affective states at the group level in the initial affect discrimination analysis. Therefore, the final resulting map shows voxels where both (i) classification was above chance across all subjects and (ii) classification varied significantly with the empathy scores.

### Statistical thresholding

To determine an appropriate threshold for significance, we employed two parallel methods to correct for multiple comparisons in each of our analyses. For the affect discrimination analysis, we first used permutation testing to create a null distribution of classification accuracies within a simulated searchlight sphere by shuffling affect labels. We ran 151 801 permutations of the classification within a single-sample searchlight sphere. This allowed us to create a distribution of classification values that might occur in a given searchlight sphere assuming the null hypothesis that the four affect conditions cannot be distinguished from patterns of activation. To account for multiple comparisons in the correlation analysis, we first performed a resel-wise Bonferroni correction: we determined the total number of five voxel radius independent spheres, which could fit in the standard brain (∼725) and divided 0.05 by this number to determine our alpha (6.89 × 10^−5^) (Kaplan and Meyer, 2012). Therefore, a classification accuracy for which greater than or equal values appeared in our distribution less than 10 times would be considered significantly above chance. In our simulated null distribution, where there were four emotions and an expected chance accuracy of 0.25, a significant above chance classification accuracy was determined to be >0.30592. The maximum value found in our null distribution was 0.311 (Supplementary Figure S2). For the second correction method in both analyses, we used a voxel-wise Bonferroni correction (0.05/*n* tests) and determined the corresponding accuracy value using the binomial distribution. With this method, we established that a Bonferroni-corrected significance of 0.05 would correspond to an accuracy threshold of 0.3815. To enhance the replicability of our findings and isolate the most informative regions, we opted in both analyses to use the more conservative threshold (permutation-corrected results can be found in the appendix: Supplementary Figure S3). This threshold is extremely conservative given that it is based on a full voxel-wise Bonferroni correction and is also greater than any of the classification results in our over 150 000 permutations.

In *post-hoc* analyses, we wanted to determine if our results in the regions that significantly distinguished between the four emotions were driven by one emotion being uniquely distinguishable compared to the others. To do this, we ran pair-wise searchlight analyses classifying each of six possible pairs of emotions. If one specific emotion was driving our findings, only pairs with that one emotion would show a similar spatial pattern of significant classification to our initial four-way searchlight emotion discrimination analysis.

## Results

Maps of the statistical results for this study can be found on the Open Science Foundation website for this study, https://osf.io/8wcnz/.

### Affect discrimination searchlight

**Fig. 1.**
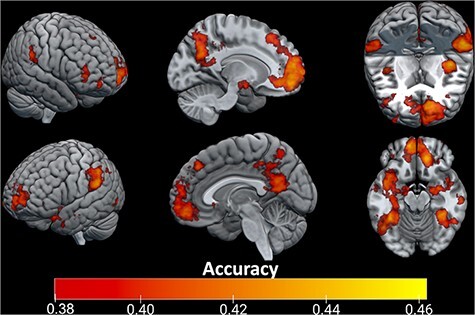
Brain regions in which the searchlight analysis significantly predicted the four affect categories. After Bonferonni correction for every voxel in the brain, a corrected *P*-value of 0.05 corresponded to a classification accuracy threshold of ∼0.38.

Multiple regions significantly predicted affect classification, even using the more conservative (voxel-wise Bonferroni-corrected) threshold (compared with the four-way chance level of 0.25) (See Figure 1). These included vmPFC (*x* = −9, *y* = 55, *z* = −16; accuracy = 0.444), anterior prefrontal cortex (*x*= −9, *y* = 55, *z* = 12; accuracy = 0.436), dorsomedial prefrontal cortex (*x* = 14, *y* = 36, *z* = 46; accuracy = 0.419), bilateral insula (left *x* = −40, *y* = −11, *z* = 9; accuracy = 0.441, right *x* = 44, *y* = −6, *z* = 8; accuracy = 0.420), bilateral amygdala (left *x* = −28, *y* = −10, *z*= −12; accuracy = 0.412; right *x* = 26, *y* = −10, *z*= −12; accuracy = 0.417), posterior cingulate cortex (*x* = −2, *y* = −52, *z* = 14; accuracy = 0.425), bilateral temporal gyrus (*x* = −38, *y* = −54, *z* = −8; accuracy = 0.446) (*x* = 33, *y* = −43, *z* = −10; accuracy = 0.448) and bilateral superior parietal lobule (left *x* = −32, *y* = −69, *z* = 37; accuracy = 0.428; right *x* = 33, *y* = −63, *z* = 30; accuracy = 0.426).

These spatial patterns, especially the high classification values in medial frontal and parietal areas, were mirrored in all the *post hoc* pair-wise classification searchlights except happy *vs* sad, which did not reach a significant threshold of 0.68 accuracy (see Supplementary Figures S4–S8). This suggests that our four-way classification results did not result from one emotion being distinguishable compared to the rest, or one pair of emotions being especially distinguishable compared to other combinations.

### Classification accuracy and IRI measures

**Fig. 2.**
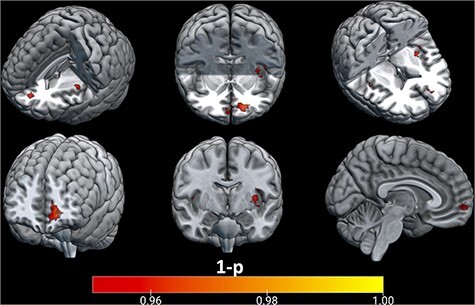
Brain regions with significant classification accuracy where PT on the Interpersonal Reactivity Index significantly predicted classifier accuracy.

**Fig. 3.**
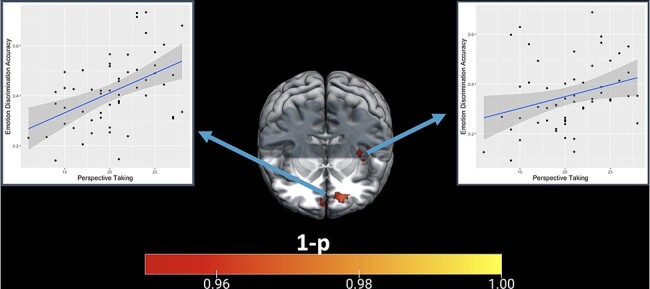
Scatterplots of PT *vs* classification accuracy in the ventromedial prefrontal cortex and insula.

See Supplementary Table S4 and Supplementary Figure S9 for descriptive statistics and distributions of sub-scales. FS, PD and EC were not significantly related to classification accuracy. PT was significantly related to classification accuracy (*P*  < 0.05 with FWE correction) in a large area of vmPFC (*x* = −15, *y* = 48, *z* = −7), as well as in bilateral, although predominantly left, insula (*x* = −39, *y* = −7, *z* = 7) (*x* = 43, *y* = −5, *z* = −6; see Figures 2 and 3).

## Discussion

MVPA has become a popular method in affective neuroscience over the past decade (Coutanche, 2013; Morawetz *et al.*, 2016; Oosterwijk *et al.*, 2017). While it has been demonstrated repeatedly that neural patterns associated with affective states can be distinguished from each other, the interpretation of what these patterns tell us about categories, and how they come to be, is contentious (for review: Coutanche, 2013; Kragel and LaBar, 2016; Clark-Polner *et al.*, 2017; Gessell *et al.*, 2021). In our study, we used a novel approach to increase our power of inference in these often ‘black-box’ like scenarios. We demonstrate that an individual trait may be related to differences in the distinguishability of neural patterns corresponding to affective states. This method provides a new way of exploring and theorizing why neural patterns can be distinguished in this way and demonstrates a new avenue of inference for testing how personal traits and cognitive functions might be associated with how different neural states are discernible from each other. In this case, we found that self-reported PT is related to the discernibility of the affective states our participants were asked to imagine. Our study has implications for both the use of MVPA in general and our understanding of how empathetic traits relate to affect.

### Relating MVPA performance to individual differences

It has long been recognized that connecting an MVPA result directly to some measured aspect of the underlying behavior or psychology being studied is important (Raizada and Kriegeskorte, 2010; Naselaris *et al.*, 2011). Otherwise, successfully decoded neural signals may reflect information that is either not used at all by the brain or is not relevant to aspects of the psychology in question (Kriegeskorte and Douglas, 2019; Ritchie *et al.*, 2019). Nevertheless, it is uncommon for MVPA studies to relate classifier performance to individual differences. Using procedures similar to ours, Coutanche *et al.* (2011) found that the MVPA classification of visual stimuli correlated with Autism Spectrum Disorder symptom severity across subjects. Studies in the auditory domain have found that MVPA classification accuracy in a musical instrument identification task (Ogg *et al.*, 2019) and a speaker identification task (Bonte *et al.*, 2014; Aglieri *et al.*, 2021) correlate with between-subject differences in accuracy on those tasks. Raizada *et al.* (2010) found that the performance of a classifier at distinguishing neural patterns related to the perception of different phonemes was related to subjects’ performance in discriminating the same phonemes and that the separability of neural patterns during a numerical discrimination task was related to arithmetic performance. Similarly, Meyer *et al.* (2010) showed that classifier discrimination of imagined auditory stimuli was correlated with the reported vividness of imagination.

Yet, while linking MVPA performance to individual differences can make headway in showing the psychological relevance of classification performance, many of the limitations that exist in other studies attempting to relate individual differences to the neuroimaging analysis still remain. For example, to optimize studying individual differences, the states induced should produce enough variability across individuals while still maintaining high identifiability of stimulus representations (Finn *et al.*, 2017). In terms of identifiability of neural patterns, MVPA tends to be more sensitive than the univariate analysis. However, it also tends to be less sensitive to between-subject variability in mean activation levels (Davis *et al.*, 2014). It remains to be determined whether the variability across people in classifier performance is optimal for the study of individual differences. Furthermore, while the trait relevance of classifier performance is informative, interpretation must always be careful in that such relationships can always be mediated by additional, unmeasured variables.

### Decoding of affective states correlates with PT

We found multiple brain regions whose activity permitted us to robustly classify evoked affective states. Many of these brain regions have allowed successful classification of affect in prior studies: the posterior cingulate, insula, temporal gyrus, medial prefrontal and ventral medial prefrontal regions have all been implicated in previous MVPA studies of emotions and affective context (Skerry and Saxe, 2014; Saarimaki *et al.*, 2016; Oosterwijk *et al.*, 2017; Bush *et al.*, 2018; Paquette *et al.*, 2018; Sachs *et al.*, 2018).

The vmPFC has long been implicated in implementing emotion and feeling (for review: Bechara *et al.*, 1994, 1999; Roy *et al.*, 2012; Winecoff *et al.*, 2013). A theoretical link between affect and the vmPFC is provided by the ‘somatic marker hypothesis’ (Damasio, 1996). This view posits that the vmPFC is a key region for incorporating emotion-related signals from the body, consciously experienced as feelings, into our cognitive decision-making process. According to the SMH, the vmPFC also permits us to bypass pure bodily input in order to simulate these feelings even without the direct presence of a trigger (Poppa and Bechara, 2018). By this account, the vmPFC could serve to ‘simulate’ the affective experience being evoked (Keysers and Gazzola, 2007; Schacter *et al.*, 2017). Indeed, studies on affective PT *vs* cognitive PT have implicated the vmPFC (Sebastian *et al.*, 2012; Healey and Grossman, 2018). The vmPFC has also been implicated in affective simulation in studies in which participants imagined affective contexts that could happen to them in the future (D’Argembeau *et al.*, 2008) or that have happened in the past (Benoit *et al.*, 2016, 2019). Correspondingly, lesions to vmPFC appear to hamper both the ability to simulate future affective scenarios and imaginary ones (Bertossi *et al.*, 2016, 2017).

Importantly, we show here that the vmPFC’s role in affective PT is related not just to the level of the neural activity invoked, but to the distinctiveness of its affect-related neural patterns. This could suggest that affective PT may relate to the simulation of the affective experience that is more specific to the target emotion, reflected functionally in more easily classifiable representations of affective context. If simulated affective states are more generalized and overlapping, as opposed to detailed and nuanced, their associated neural patterns can be expected to be less differentiable. Perceived emotional actions, interoception and affective contexts of other individuals can all be successfully classified using the vmPFC activity (Oosterwijk *et al.*, 2017). Notably, areas where accuracy was significantly associated with PT showed little overlap with regions typically implicated in univariate studies of PT: regions such as visual cortex, temporal–parietal junction and dorsolateral prefrontal cortex (Decety and Grèzes, 2006; Bukowski, 2018; Healey and Grossman, 2018). Accuracy in these areas did not correlate with our PT measure. Additionally, even though it is conceivable that our visual and descriptive prompts would support a largely visual simulation of the scene or that improved affective representation might result from increased attention, neither primary visual regions nor parietal and temporal visuospatial regions provided significant classification accuracy. Visual, temporal–parietal and dorsal prefrontal regions may be important for the general process of PT but more related to the general effort involved in performing the task rather than its success. In a study where individuals were instructed to take the perspective that an image of a painful stimulus was occurring to either themselves or another person (van der Heiden *et al.*, 2013), the main effects of the different PT conditions were evident in regions such as supramarginal gyrus, temporal gyrus, frontal gyrus and ventrolateral PFC. However, when the effects of good *vs* bad perspective-takers were analyzed, all these regions except ventrolateral PFC were not significant predictors, and instead the left insula, postcentral gyrus and vmPFC differed between the groups. Perhaps the insula and vmPFC are specifically important for generating a fine-grained affective simulation, and this granularity is reflected in more distinct neural patterns.

We note also that while the IRI includes items that ask participants to judge their ease or difficulty at empathizing (‘I sometimes find it difficult to see things from the “other guy’s” point of view’.) many of the questions are instead about the ‘tendency’ to take someone else’s perspective (‘I sometimes try to understand my friends better by imagining how things look from their perspective’.) or the ‘motivation’ to do so (‘I try to look at everybody’s side of a disagreement before I make a decision’.). As such, we cannot distinguish which aspects of PT are directly related to increased classifier accuracy. It is possible that participants who score higher on the IRI-PT are motivated to engage in the task with more effort and that the increased pattern separation is related to increased effort in the task. Indeed, the IRI-PT is correlated with the Mind Reading Motivation scale, a measure specifically aimed at the motivation to think about other people’s minds (Carpenter *et al.*, 2016).

### Future directions

Our searchlight analysis found large regions of the brain, many previously implicated in affect-related processing, that significantly classified affective states. Despite this, only the accuracy in the vmPFC and insula was related to PT. This leaves the question of what traits, cognitive processes, or noise factors could potentially explain individual differences in classification accuracy in other regions. It is possible that other cognitive traits, such as the ability to interpret other’s affective intent or bodily perception, may explain how accurate other regions such as temporal gyrus (Wicker *et al.*, 2003) and the parietal lobule (Engelen *et al.*, 2015) are at distinguishing affective states in MVPA studies. Furthermore, the affective states we asked participants to imagine are among the most common, ‘basic emotions’. These affective experiences are most commonly described as categorical in daily life and therefore may be more easily discriminated. It remains unclear whether the same mechanisms, PT and affective simulation in the vmPFC, would be as accurate in discriminating mixed feelings or if other neural regions and cognitive processes may be differentially important for non-typical affective states.

Our study highlights the importance of investigating the neural substrate of individual differences in PT overall, especially across domains. While some brain regions may be involved broadly in PT across participants, univariate approaches may miss more specific regions that truly distinguish successful *vs* unsuccessful PT. In our affective simulation task, these were the vmPFC and insula, although these likely may be different for PT tasks involving other domains.

## Conclusion

In our study, we found a relationship between trait PT ability and the classification accuracy of an individual’s vmPFC and insular activity for distinguishing task-evoked affective states. The value of these findings is important because it shows that the discriminability of signal in these regions, which exhibit high classification accuracy among affective states overall, is associated with a task-relevant personal trait, namely PT. More work is needed, however, to explore what underlying functional properties underlie successful classification (Anderson *et al.*, 2012; Carlson and Wardle, 2015).

Methodologically, we show that searchlight MVPA can be used to uncover the mediating traits and processes, which explain why a particular region of the brain contributes to classification accuracy. Connecting MVPA results directly to individual traits or behaviors greatly enhances their interpretability. These findings reflect the strength of both multivariate analysis and the study of individual differences: both seek to gain information from what ‘differs between’ participants, rather than averages and commonalities across participants.

## Supplementary Material

nsac035_Supp
