# Supplementary material for: Perspective-taking is associated with increased discriminability of affective states in the ventromedial prefrontal cortex
Source: Soc Cogn Affect Neurosci. 2022 May 17;17(12):1082–90. doi: 10.1093/scan/nsac035 (PMC9714424; doi:10.1093/scan/nsac035)
Supplement: nsac035_Supp [file nsac035_supp.zip › Supplementary Material.docx]

**Supplementary Material**

Table S1: Examples of Stimuli Sentence-Picture Pairs

| **Stimuli** | **Category** | **Image Description** | **IAPS or ANET ID** |  |  |
| --- | --- | --- | --- | --- | --- |
| 1 | Happy | man jumping for joy in store | ANET_8500 |  |  |
| 2 | Happy | child laughing at table with family | ANET_2560 |  |  |
| 3 | Fear | dog with teeth bared | IAPS 1301 |  |  |
| 4 | Fear | robber in a mask | ANET_6370 |  |  |
| 5 | Disgust | bug on slice of pizza | IAPS 7380 |  |  |
| 6 | Disgust | man throwing up on other man | IAPS 9321 |  |  |
| 7 | Sad | old man with dying woman | IAPS 2205 |  |  |
| 8 | Sad | woman and man crying at factory | IAPS 2456 |  |  |
| 9 | Neutral | grocery store aisle | ANET_2520 |  |  |
| 10 | Neutral | flashlight shining on map | ANET_7040 |  |  |
| **Stimuli** | **Sentence** |  |  |  |  |
| 1 | You've just won 10 million dollars; you jump up and down, screaming. | | |  |  |
| 2 | You lounge around the crowded table, laughing with your family. | | |  |  |
| 3 | The dog strains forward, snarling, and suddenly leaps out at you. | | |  |  |
| 4 | You freeze as a masked figure looms over you and ties you to the bed. | | |  |  |
| 5 | You gag, seeing a roach moving slowly over the surface of the pizza. | | |  |  |
| 6 | As you leave the concert, a drunk vomits all over your jacket, soaking it. | | |  |  |
| 7 | After fifty years of marriage, your wife has died, leaving you alone. | | |  |  |
| 8 | Today you found out you lost your job, and you won't be able to buy food for your children. | | |  |  |
| 9 | You walk through the grocery aisles, adding necessary items to your cart. | | |  |  |
| 10 | You hold the flashlight steady in order to get a better look at the map. | | |  |  |

Table S2: Mean (Standard Deviation) of Rated Valence and Arousal of Stimuli (1-7 scale)

| **Emotion** | Valence | Arousal |
| --- | --- | --- |
| Happy | 6.94 (.633) | 5.09 (.844) |
| Fear | 2.96 (1.10) | 6.81 (.541) |
| Disgust | 2.72 (1.01) | 5.70 (.994) |
| Sad | 2.67 (.665) | 4.72 (.557) |
| Neutral | 5.49 (.892) | 4.49 (.590) |

Table S3 T-values From Two Sample T-Tests Comparing Valence and Arousal of Stimuli

| **Valence** | Happy | Fear | Disgust | Sad | Neutral |
| --- | --- | --- | --- | --- | --- |
| Happy | x | 10.852** | 12.231** | 16.094** | 4.593** |
| Fear | x | x | 0.551 | 0.794 | -6.174** |
| Disgust | x | x | x | 0.164 | -7.093** |
| Sad | x | x | x | x | -8.768** |
| Neutral | x | x | x | x | x |
| **Arousal** | Happy | Fear | Disgust | Sad | Neutral |
| Happy | x | -5.937* | -1.602 | 1.27 | 2.037 |
| Fear | x | x | 3.413* | 9.232** | 10.059** |
| Disgust | x | x | x | 2.961* | 3.623* |
| Sad | x | x | x | x | 1.002 |
| Neutral | x | x | x | x | x |

*p<.01

**p<.001

Table S4: Average Number of Participants Choosing Stimuli Category by Category of Emotion

|  | **Chosen Category** | |  |  |  |
| --- | --- | --- | --- | --- | --- |
| **Intended** | Happy | Fear | Disgust | Sadness | Neutral |
| Happy | 30.33 | 0.58 | 0.42 | 0.75 | 11 |
| Fear | 2.75 | 27.67 | 1.5 | 6.08 | 5.17 |
| Disgust | 1.83 | 4 | 31.67 | 8.5 | 5.67 |
| Sadness | 0 | 6 | 6 | 32 | 6 |
| Neutral | 14.67 | 1 | 1.5 | 1.42 | 26.42 |
| **Intended** | Anger | Anxiety | Shame | Love | Surprise |
| Happy | 0.33 | 1.75 | 0.42 | 15.83 | 5.66 |
| Fear | 3.08 | 24.58 | 0.5 | 0.25 | 6.67 |
| Disgust | 2.17 | 8 | 3.67 | 1.33 | 2.17 |
| Sadness | 12 | 19 | 4 | 1 | 0 |
| Neutral | 0.67 | 5.58 | 1.75 | 2.83 | 1 |

Table S5: Mean and Standard Deviations of Empathy Sub-scales

| **IRI Sub-Scale** | Mean | Standard Deviation |
| --- | --- | --- |
| Empathic Concern | 20.31 | 4.118 |
| Perspective Taking | 20.42 | 4.18 |
| Personal Distress | 10.8 | 4.266 |
| Fantasy | 18.24 | 4.823 |

**Stimuli Presentation**

Subjects were given the following instructions: “When the experiment begins, you will see a series of pictures and words. Each picture will appear on the screen in front of you with a short sentence below it. Please read the statement, look at the picture, and try to feel the emotions and feelings described by the scenario as strongly as possible. The picture will stay on the screen for several seconds. When the picture disappears, do your best to put it out of your mind. After a short period, the next picture will appear.”

**Comparison of individual differences in subjects from study 1 and study 2**

Since the 36 subjects from the study 1 data had 4 functional runs, while the 19 subjects from study 2 had 3 functional runs, we ran an analysis to compare the classification accuracy between the two groups. This way, we can determine if our findings are due to different numbers of trials across our dataset. To compare individual differences in classification accuracy between 3-run and 4-run subjects, we used FSL’s Randomize tool to compare the searchlight maps of both cohorts of subjects. We then tested the overlap between these voxels that differed and the voxels in our main result: the voxels significant for perspective-taking correlating with emotion discrimination accuracy in regions where emotion discrimination accuracy was significant overall.

81% of voxels that show correlation between perspective-taking and accuracy did not significantly differ between subjects with three trials and subjects with four trials. Furthermore, we have tested for differences in perspective taking scores between the two groups, and found no difference (M1=20.89 M2=20.17, p=.54). These show that our findings are not due to the varying number of trials or differences in perspective-taking between the two groups of participants.

**Figure S1: Proportion of participants that rated emotion stimuli in category**

**Figure S2: Distribution of null classification accuracy**

**Figure S3: Accuracy of emotion classification searchlight using the minimum threshold derived from permutation testing**

**Figure S4: Searchlight of Happy vs. Fear**

**Figure S5: Searchlight of Happy vs. Disgust**

**Figure S6: Searchlight of Sad vs. Fear**
 **Figure S7: Searchlight of Sad vs. Disgust**

**Figure S8: Searchlight of Fear vs. Disgust**

**Figure S9: Distributions of Interpersonal Reactivity Index Sub-scales**
