# Supplementary figures and images for: Perspective-taking is associated with increased discriminability of affective states in the ventromedial prefrontal cortex
Source: Soc Cogn Affect Neurosci. 2022 May 17;17(12):1082–90. doi: 10.1093/scan/nsac035 (PMC9714424; doi:10.1093/scan/nsac035)

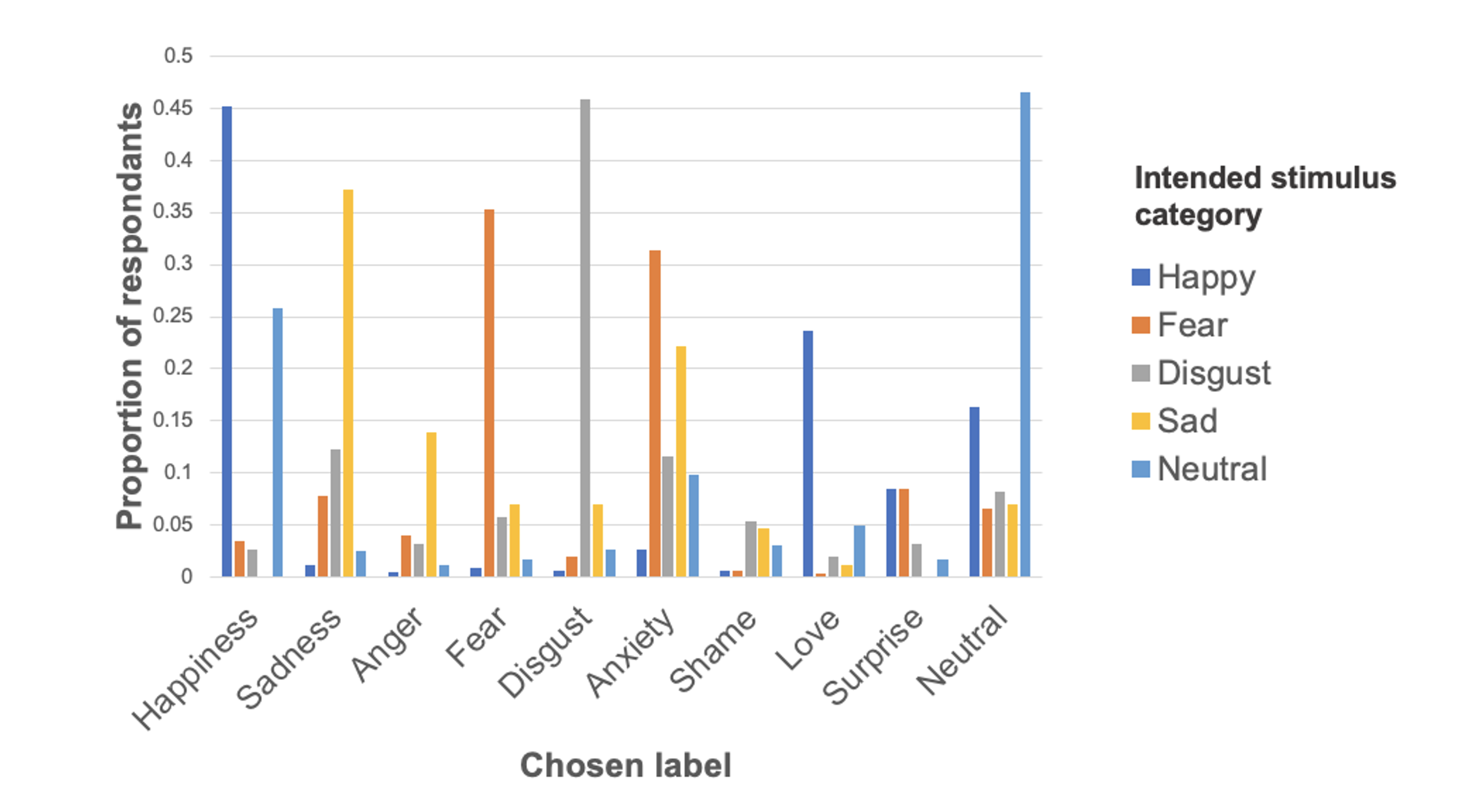

Supplement: nsac035_Supp [file nsac035_supp.zip › scan-21-071-File006.tiff]

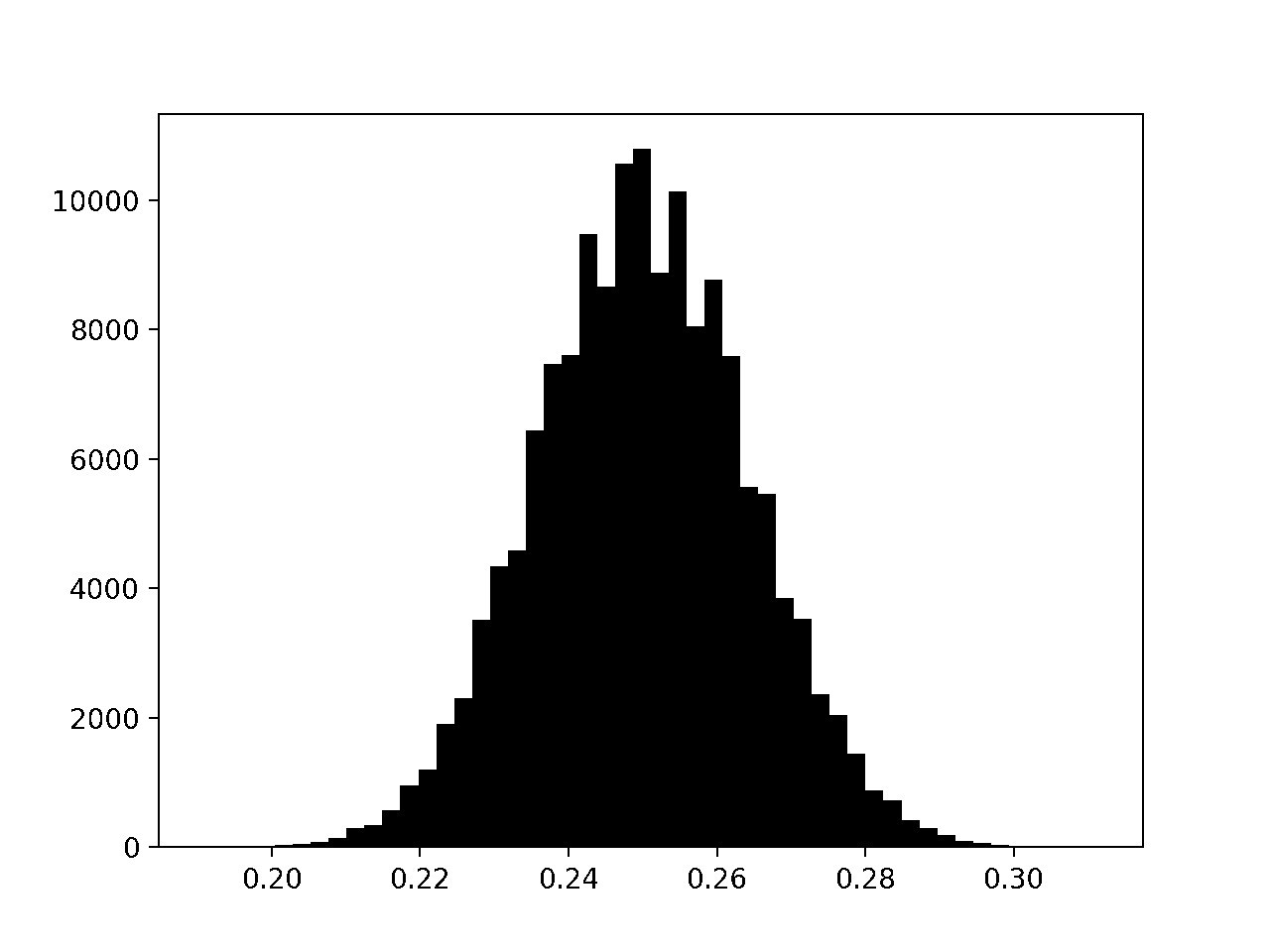

Supplement: nsac035_Supp [file nsac035_supp.zip › scan-21-071-File007.tiff]

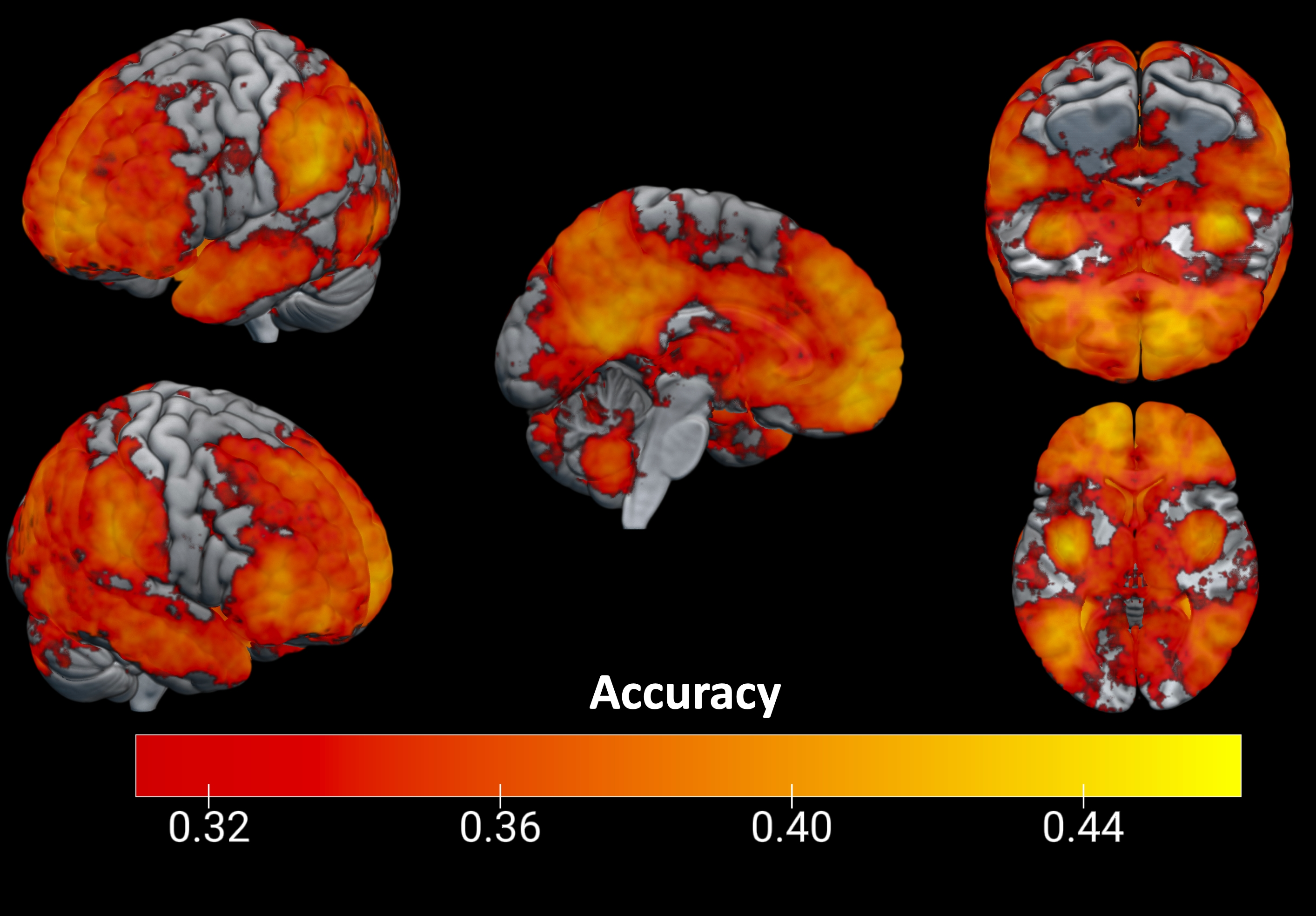

Supplement: nsac035_Supp [file nsac035_supp.zip › scan-21-071-File008.tiff]

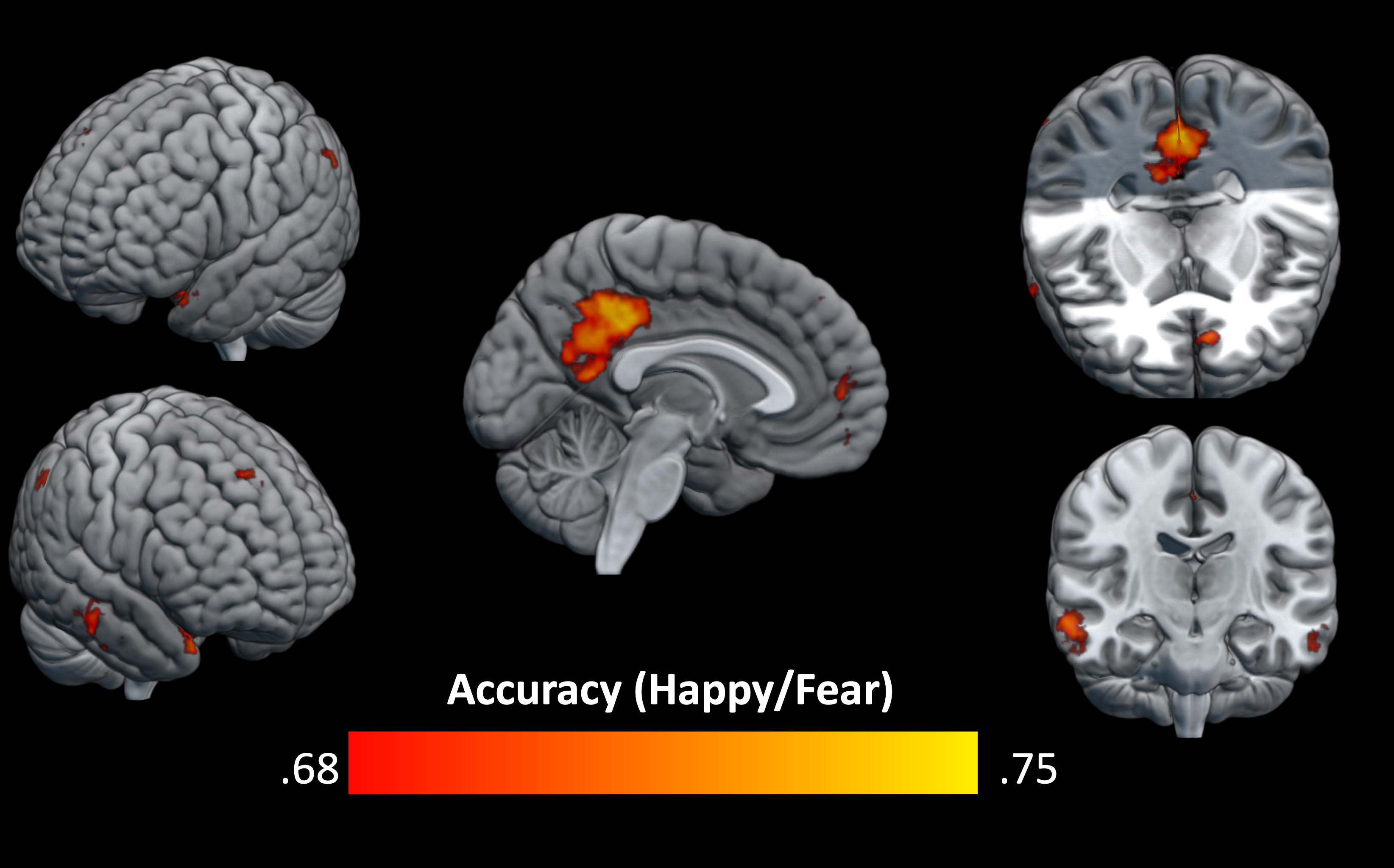

Supplement: nsac035_Supp [file nsac035_supp.zip › scan-21-071-File009.tiff]

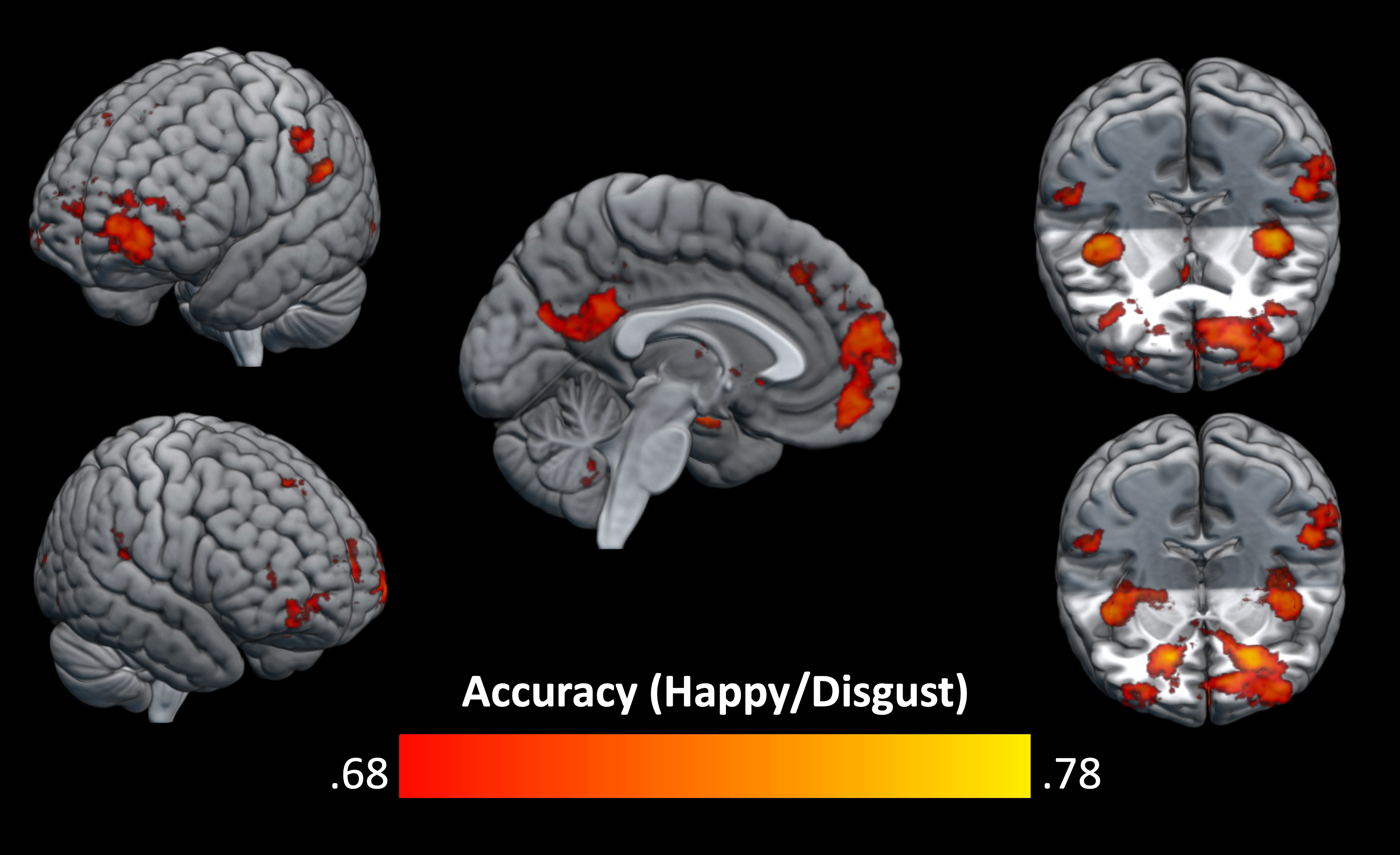

Supplement: nsac035_Supp [file nsac035_supp.zip › scan-21-071-File010.tiff]

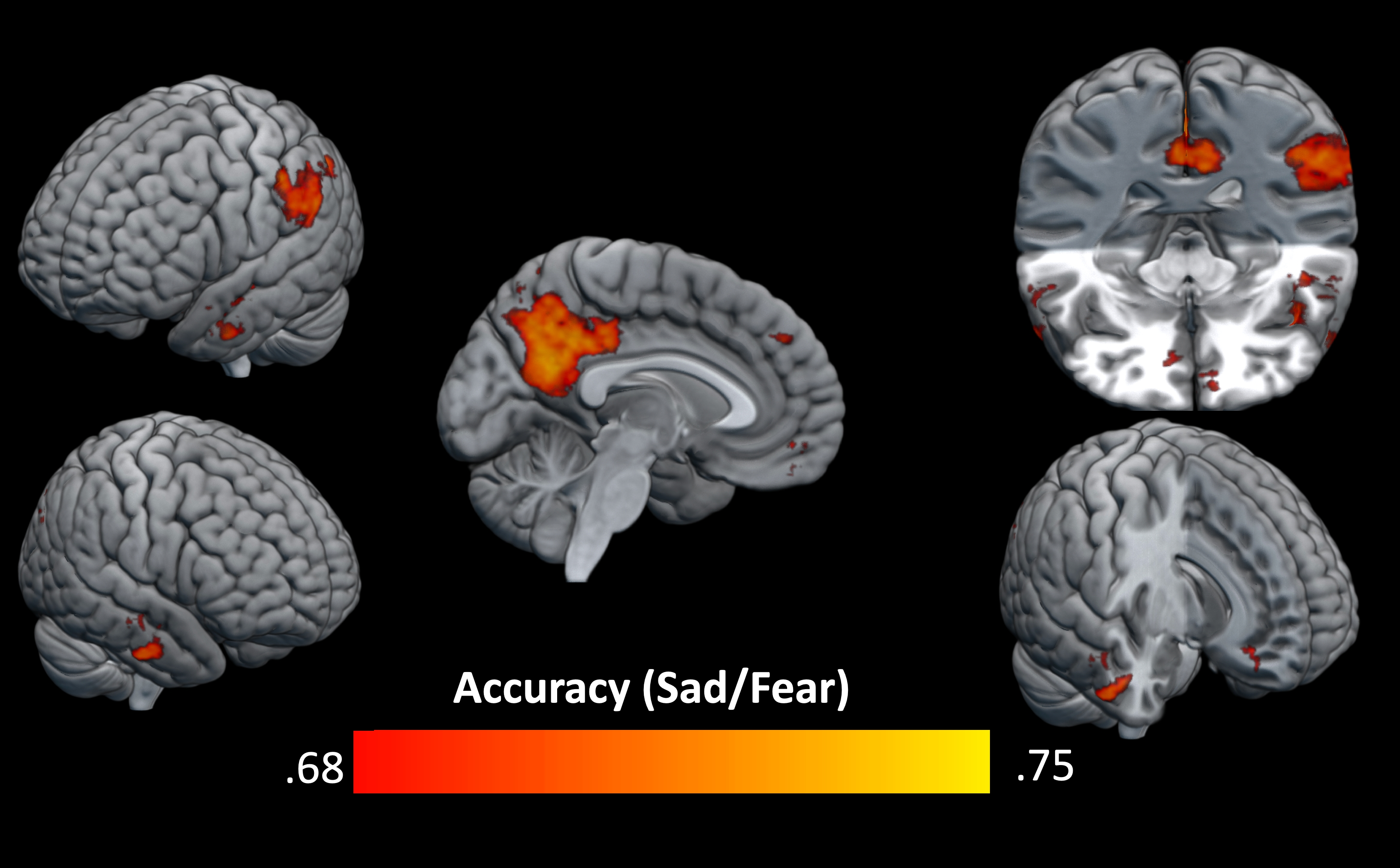

Supplement: nsac035_Supp [file nsac035_supp.zip › scan-21-071-File011.tiff]

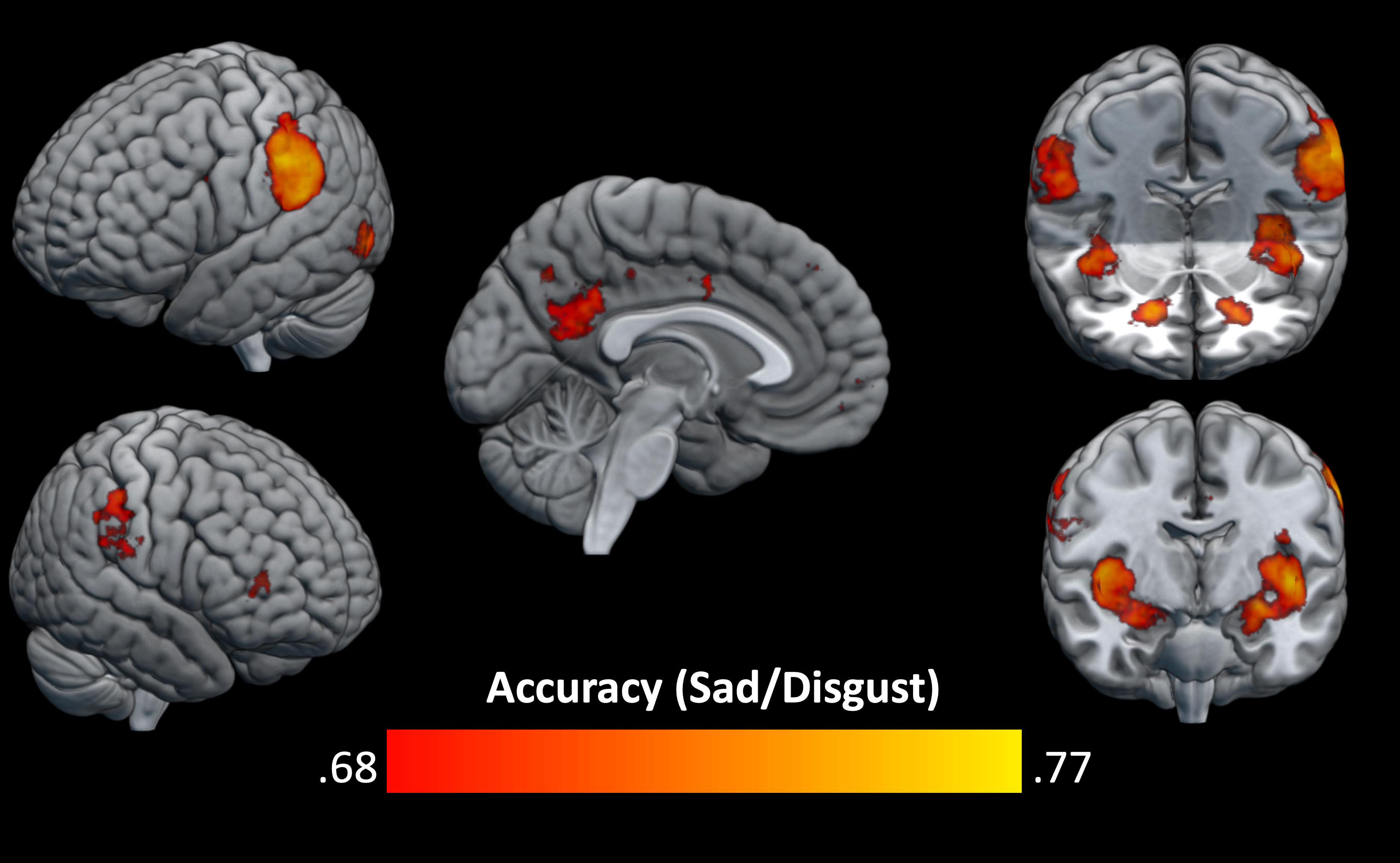

Supplement: nsac035_Supp [file nsac035_supp.zip › scan-21-071-File012.tiff]

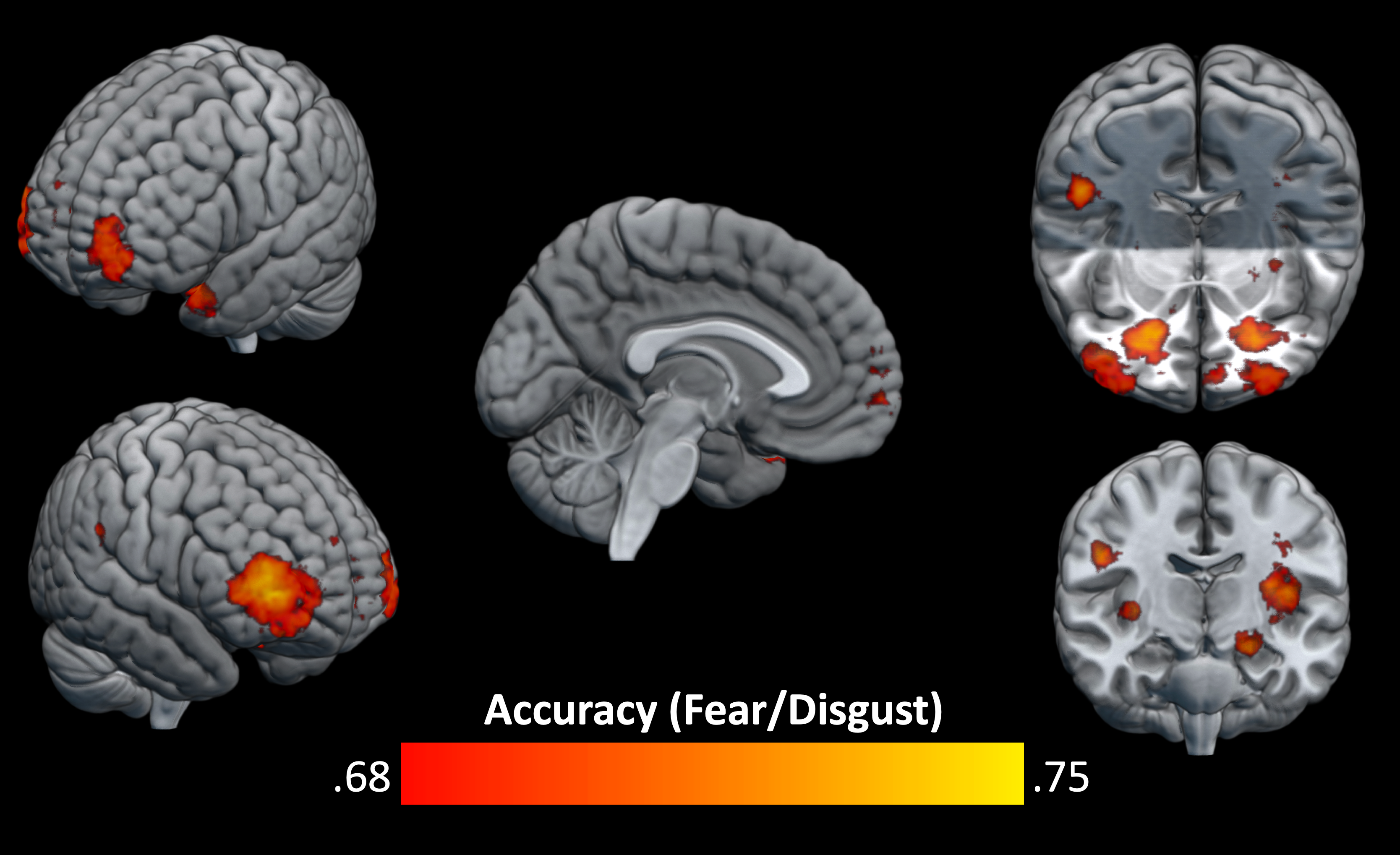

Supplement: nsac035_Supp [file nsac035_supp.zip › scan-21-071-File013.tiff]

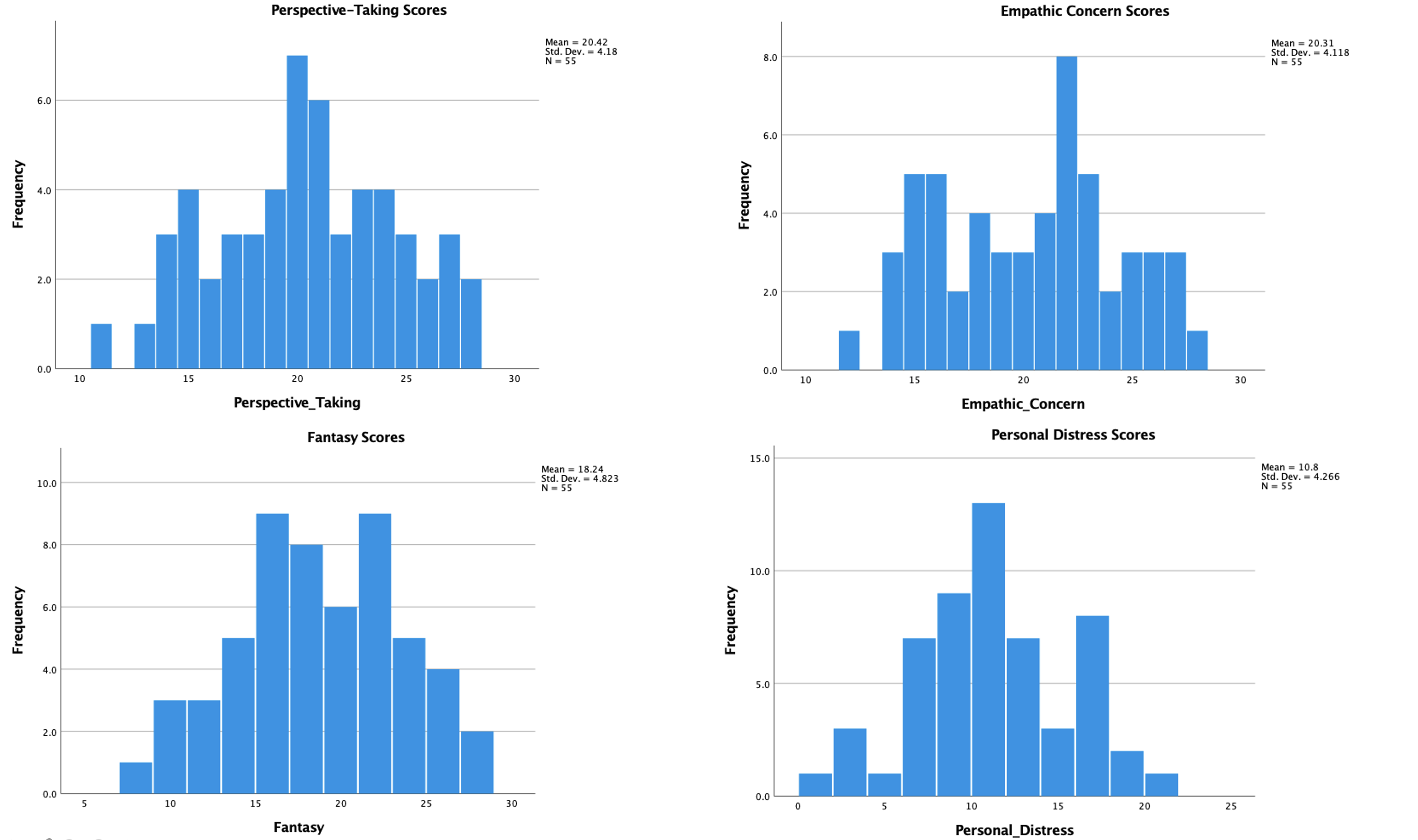

Supplement: nsac035_Supp [file nsac035_supp.zip › scan-21-071-File014.tiff]
